# Malaria and Travel to the Dominican Republic

**DOI:** 10.3201/eid1103.040898

**Published:** 2005-03

**Authors:** Juan L. Haro-González, Máximo Bernabeu-Wittel, Elías Cañas, Carmen Regordán

**Affiliations:** * Hospitales Universitarios Virgen del Rocío, Seville, Spain

**Keywords:** Dominican Republic, malaria, *Plasmodium vivax*, *Plasmodium malariae*

**To the Editor:** The rise in international travel to malaria-endemic areas in recent years has been followed by an increase in the number of cases diagnosed in countries where malaria is not endemic (1). Tourist areas of the Dominican Republic have traditionally been considered to be low risk for malaria transmission. However, over the past few years, sporadic descriptions of imported falciparum malaria in travelers to these destinations have been described (2,3). In spite of these findings, neither the World Health Organization nor the Centers for Disease Control and Prevention recommend antimalarial chemoprophylaxis for trips to the Dominican Republic's main tourist resorts (4,5).^1^ We report a new case of imported malaria caused by mixed *Plasmodium vivax* and *P. malariae* infection, with unique clinical features, after a standard tourist trip to Puerto Plata (on the northern coast of the Dominican Republic).

A 31-year-old man with no relevant medical history was treated in the internal medicine department of our hospital. He reported a history of poorly defined malaise, night sweats, sleeplessness, tinnitus, and episodic diarrhea with no pathologic products during the previous 6 days. He did not report fever, chills, or headache. Two weeks earlier, he had spent 10 days in Puerto Plata in a tourist resort, without traveling to any other place. He had not received any antimalarial chemoprophylaxis. Physical examination showed no abnormalities. Laboratory values, including levels of sodium, potassium, liver enzymes, creatinine, and coagulation factors, as well as results of hemogram and chest radiograph, were within normal limits. A blood film showed trophozoytes of *P. vivax* and *P. malariae*. In a stool specimen, *Entamoeba histolytica*, *Trichiuris trichura*, *Endolimax nana*, and *Blastocystis hominis* were observed; stool cultures were negative.

Treatment was initiated with chloroquine (4 doses) and primaquine for a period of 14 days; metronidazole and paromomycin were administered for the intestinal infestations. Symptoms resolved in 48 hours, and control blood films showed clearance of the parasitemia. Two months after the end of treatment, the patient remained asymptomatic.

We describe a new and unusual case of imported vivax-malariae malaria. Two characteristics of our patient's case bear mention. First, the place of acquisition of the infection and the species of *Plasmodium* involved are notable. The Dominican Republic is considered a low-risk area for malaria, although some places in the west, on the Haitian border, are malaria-endemic. In addition, according to available information, autochthonous malaria cases increased after Hurricane George (3,003 cases in 1999, compared to 2,000 in 1998) (6). Previously described sporadic cases of imported malaria from the Dominican Republic included those in tourists who traveled to Punta Cana, in the eastern part of the country. All these cases were caused by *P. falciparum*. To our knowledge, no cases of *P. vivax* or mixed *P. vivax*/*P. malariae* infection have been described after travel to the Dominican Republic (2,3). From January 1999 to September 2003, TropNetEurop (a European surveillance network of tropical and imported diseases) noted 618 cases of *P. vivax* infection imported to Europe. The most common areas of acquisition of *P. vivax* infection were the Indian subcontinent (17%), Indonesia (12.1%), South America (11.4%), and West Africa (11.4%). Only 0.2% of the cases of *P. vivax* infection were acquired in the Caribbean, none of them in the Dominican Republic (7).

Second, the clinical features were atypical. Malaria usually starts as a febrile syndrome, accompanied by chills, headache, malaise, and arthromyalgia. However, sometimes symptoms are unspecific. In fact <10% of patients do not exhibit fever or chills, and some report only poorly defined complaints or other atypical symptoms. Among these, gastrointestinal symptoms are the most frequently reported (8). In the present case, the syndrome could have been easily explained by the intestinal infestations detected in stool studies, and malaria would have been overlooked if the clinician had not taken into account this disease in the diagnostic workup.

In summary, clinicians should include malaria in the diagnostic workup of tourists who become ill after traveling to the Dominican Republic. Species other than *P. falciparum* may be the cause of the disease; these species likely induce more atypical forms of malaria.

## Footnotes

1 At time of publication, neither WHO nor CDC made recommendations. For current recommendations, see http://www.cdc.gov/travel/regionalmalaria/caribean.htm
